# Comparison of the Haas Expander and the Elastodontic Device for the Resolution of Transverse Discrepancies in Growing Patients: A Single-Centre Observational Study

**DOI:** 10.3390/reports7020041

**Published:** 2024-05-21

**Authors:** Eleonora Ortu, Sara Di Nicolantonio, Samuele Cova, Davide Pietropaoli, Lucia De Simone, Annalisa Monaco

**Affiliations:** 1 MeSVA Department, Dental Unit, University of L’Aquila, P.le S. Tommasi, 67100 L’Aquila, Italy; eleonora.ortu@univaq.it (E.O.); davide.pietropaoli@univaq.it (D.P.); lucia.desimone@graduate.univaq.it (L.D.S.); annalisa.monaco@univaq.it (A.M.); 2 Independent Researcher, Cles, 38023 Trento, Italy; covasamuele@hotmail.com

**Keywords:** elastodontic device, intraoral scanning, palatal expansion

## Abstract

Background: This study aimed to compare the clinical outcomes of using two different devices to treat upper palatal discrepancies evaluated with a digital intraoral scanner. Methods: A total of 64 patients were enrolled and treated with either an elastodontic expansion device (32 patient test group, 16 females and 16 males, mean age 7.08 ± 0.44) or Haas expander (32 patient control group, 16 females and 16 males, mean age 7.32 ± 0.50). The two groups exhibited similar orthodontic features. The orthodontic criteria were: skeletal class I relationship; molar class I relationship; complete eruption of upper sixths; presence of unilateral or bilateral cross bite. All dental casts were examined and subsequently scanned with an intraoral scanner (I-Tero) pre-treatment (T0) and 12 months after the onset of therapy (T1) to assess the distance between the decidous upper canines (ICW, intercanine width) and the distance between the mesiopalatal cusps of the upper first molars (IMW, intermolar width). For statistical analysis, the *t*-test for continous variables and the chi-square test for categorical variables were used, respectively. Results: There were no statistically significant differences between the mean and SD of the expansions that resulted from the Haas expander and the elastodontic devices (Haas expander vs. Eptamed: ICW_T1 (Haas) = 42.34 (3.09), ICW_T1 (Eptamed) = 42.69 (2.77); *p* = 0.743; IMW_T1 (Haas) = 34.22 (2.29), IMW_T1 (Eptamed) = 34.00 (2.56); *p* = 0.800). The two devices were similarly effective. Conclusions: Elastodontic devices and the Haas expander can successfully help the orthodontist to conduct upper arch expansion treatment. However, elastodontic devices are more comfortable during the resolution of palatal discrepancies compared to palatal expander devices.

## 1. Introduction

The most common skeletal alterations involving the upper jaw are the transverse alterations, which always occur in relation to other alterations, including functional ones. Among these transverse alterations, the reduction in transverse distance is the most common and it is referred to as a transverse jaw deficit (TDM), which requires an orthopedic-orthodontic treatment in growing patients [1]. Several etiological factors underlie this condition, including genetic, environmental, anatomic, lingual posture, and breathing factors. Their combined effects may cause malocclusions and they are manifested by a reduction of the transverse diameter of the maxilla in association with a unilateral or bilateral cross-bite [2]. In most cases, transverse discrepancies of the upper jaw are associated with hypoplasia or asymmetry of the maxilla itself, so the treatment of choice in these cases is maxillary bone expansion [3]. Treatment of upper arch discrepancies can provide benefits not only at the dental level but also at the oropharyngeal and whole-organism levels. In effect, palatal expansion acts on the median palatine suture to increase the size of the upper arch and at the same time imparts a rotational force in the buccal direction on the maxillary alveolar shelves [4]. This technique, widely used in the past as well as today, acts on multiple structures of the skull. The patient who undergoes upper arch expansion typically exhibits pronounced and correlated whole-body complications of malocclusion [5]. A very important but incompletely characterized mechanism of action of palatal expansion is exerted at the level of the upper airway [6]. It is thought that, with a palatal expansion, there will be an increase in the size of the nasal passages, so there will be a reduction in air resistance. All this will facilitate nasal physiological breathing. Side effects of palate expanders are usually temporary and short-lived. However, they can also be more serious. Risks include: discomfort during treatment, speech changes, traumatic separation of the midpalatal suture (the central fusion of the hard palate), lack of cooperation, bite opening (a gap between top and bottom teeth when the mouth is closed), relapse (palate shifts back out of position), and root resorption (when the body’s immune system dissolves a tooth’s root, which can occur with orthodontic pressure).

In addition, it has been reported by several studies that rapid palatal expansion can also affect vision, causing cases of diplopia or strabismus because of the close anatomical connection of the palatal vault with other anatomical structures of the visual system [7]. For this reason, careful diagnostic evaluation of transverse defects is necessary. 

The first diagnostic approach to transverse discrepancies of the maxilla starts during the first consultation: the evaluation will be made by an orthodontist who will prescribe the necessary radiographic studies and will proceed to take impressions and record extra- and intra-oral photographs. The diagnostic procedure for assessing the need for maxillary expansion is to determine the transverse relationships between the dental arches. These measurements are made on plaster models, and different landmarks are taken depending on the patient’s stage of growth. In deciduous dentition, the distance in millimeters between the upper deciduous first molars is considered, while in mixed or permanent dentition, the distance in millimeters between the permanent upper first molars is considered, in addition to the intercanine distance [8].

Although orthodontists currently use many devices, the final objective is the same. Once the type and severity of transverse discrepancy of the maxilla have been diagnosed, a course of therapy can be designed. Patient age and histologic development of the medial palatine suture influence the choice of therapeutic device. Physiologic growth prevents widening of the median palatine suture, making it necessary to apply more force to perform this movement. In fact, true sutural growth stimulation is only possible in subjects who have not reached peak pubertal growth, whereas in late-growth subjects expansion occurs through micro-fractures of the sutural region [9]. It is therefore important to assess, in order to designate the best therapeutic choice, the patient’s age and thus whether or not he or she has reached peak growth, the patient’s cooperation, and the presence or absence of other functional impairments such as swallowing with lingual interposition and oral respiration. The aim of this paper was to verify any clinical changes in the width of the upper first intermolar (IMW) and upper intermolar (ICW) in growing patients using these two types of devices (EQ Series 00 [Eptamed] versus Haas expander). The Haas expander was first pioneered by Haas himself in 1961 and has always been used in clinical practice for rapid expansion of the maxillary upper jaw; it acts through a distraction of the median palatine suture, resulting in remodeling of the entire cranio-maxillofacial complex [10]. The Eptamed balancer, on the other hand, is an innovative device capable of acting by rehabilitating stomatognathic functions. The elastomeric material of which it is composed promotes orthodontic movement in synergy with the neuromyofascial system, directing the patient toward a correct growth vector [11]. 

This Equilibrator is a removable, highly elastic appliance that: avoids contact between the teeth by facilitating repositioning; avoids contact between the tongue and teeth; reactivates nasal breathing; encourages the tongue to go on the palate; and rebalances the muscles of the mouth and face [12]. The purpose of this work is to provide knowledge on the use of balancers, which exist in many sizes and degrees of hardness. These devices are individually chosen and modified by the dentist according to the needs of the individual patient. They are in fact INDIVIDUALISED devices. These modifications are a great added value, as it is almost never necessary to intervene on the patient’s teeth with occlusal elevations, but only on the appliance. Such a technique is gaining more and more acceptance in recent years, being a simple technique, with greater comfort and compliance by young patients [13]. However, there is still a shortage of studies on the therapeutic use of such devices, and our aim is to fill these gaps in the literature and try to provide new therapeutic insights.

Our hypothesis is that there are no differences between the Eptamed and Haas groups after palatal expansion. The authors of this study compared dental models before treatment and after 12 months.

## 2. Materials and Methods

**Figure 1:**
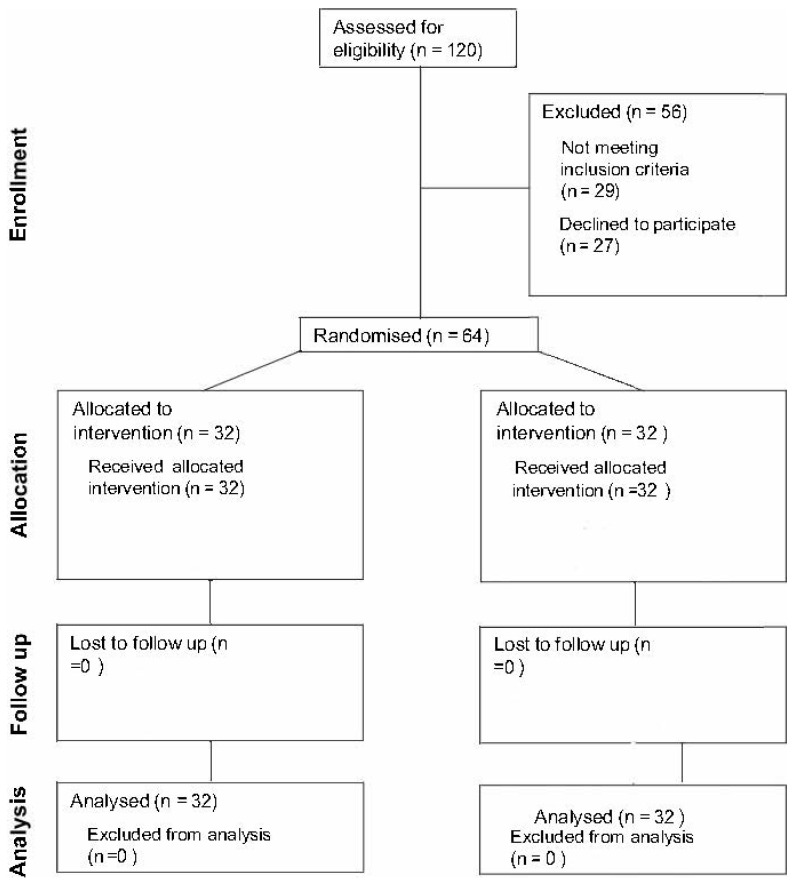
Flow diagram of study population.

This study was carried out in conformity with the basic principles of the Declaration of Helsinki. Before the study was begun, the protocol was approved by the Internal Review Board of the University Degli Studi Dell’Aquila, Italy (57/2021–22). We conducted a single-center observational study from a database (archived plaster models of the arches of patients already receiving orthodontic treatment at the Dental Clinic) to evaluate two orthodontic devices. Initially, cases of 120 patients aged 7 to 8 years treated at the Dental Clinic of the Department of Clinical Medicine, Public Health, Life and Environmental Sciences until March 2020 were reviewed. All evaluations were performed by the same clinician (AM) and included assessment of orthopantomography completed in accordance with the European guidelines on radiation protection in dental radiology; examination of intra- and extraoral photographs; and study of dental casts. From these data, the orthodontist established a specific treatment plan for every patient, according to the Indices of Need for Orthodontic Treatment (IOTN) described by Brook and Shaw (Brook and Shaw, 1989). Of these 120 patients, 27 did not give consent to participate, while 29 did not meet the inclusion criteria considered (Figure 1). Following the inclusion and exclusion criteria listed below, 64 patients were enrolled and treated with either an elastodontic expansion device (32 patient test group, 16 females and 16 males, mean age 7.08 ± 0.44) or Haas expander (32 patient control group, 16 females and 16 males, mean age 7.32 ± 0.50). 

Exclusion criteria: IOTN index > 4, presence of caries, presence of temporomandibular disorders, epilepsy, systemic diseases, periodontal disease, absence of written informed consent signed by parents/legal guardians.

Inclusion criteria: skeletal class I relationship, molar class I relationship; complete eruption of upper sixths; presence of unilateral or bilateral cross bite (falling within grade 3 IOTN index).

All plaster models were examined and then scanned with an intraoral scanner (I-Tero Element, Align Technology, San Francisco, CA, USA) pre-treatment (T0) and twelve months post-treatment (T1). All digital scans were imported into Ortho-Cad 5.9.1.50 software (3Shape A/S, Copenhagen, Denmark) to perform linear measures and 3D assessment of palate morphology at both T0 and T1. All measurements were made by the same operator. To assess the reliability and reproducibility of the measurement technique, a combined error of position, tracking, and landmark measurement was determined. The method error was calculated from the double measurement of 30 randomly selected dental models, measured again after an interval of 1 week, using Dahlberg’s formula.

**Figure 2:**
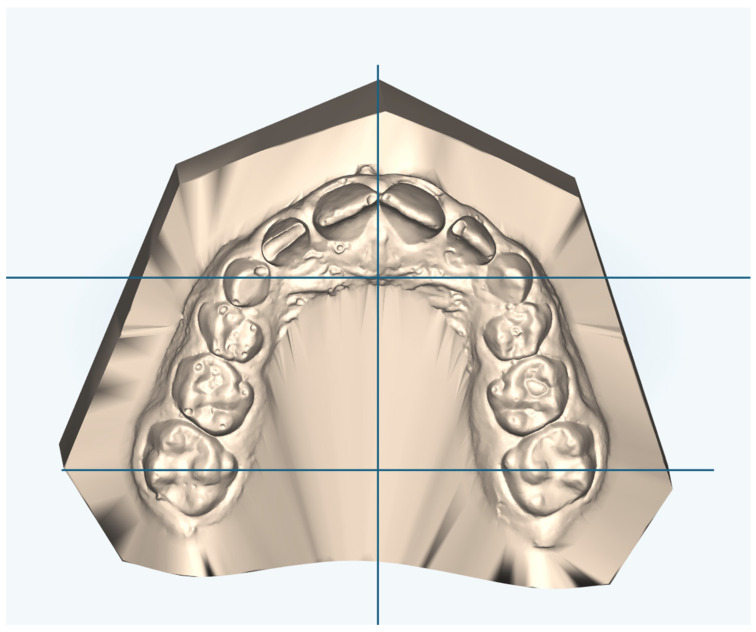
Linear measurements performed in this study to assess the transverse dimensions of the palate. Inter-canine width (ICW), inter-molar width (IMW).

First, the palatal transverse dimension was calculated at the level of the permanent first molars or deciduous ones (IMW, intermolar width) and deciduous canines (ICW, intercanine width) (Figure 2). In the group treated with elastodontic device expander, there were 10 cases of unilateral crossbite, while the remainder were bilateral. In the Haas group, there were 14 cases with unilateral crossbite.

For statistical analysis, the *t*-test for continuous variables and chi-square test for categorical variables (sex) were used. Even though the Shapiro Wilk normality test revealed normal distribution, we adopted a non-parametric Wilcoxon sum-rank test for conservativeness. Patients were divided into test and control groups in a randomized manner, through computer generated software (Sealed Envelope Ltd. 2012. Power calculator for continuous outcome superiority trial. [Online] Available from: https://www.sealedenvelope.com/power/continuous-superiority/ accessed on 24 January 2023) and were stratified by center with a 1:1 allocation using random block size of 4, 6, 8.

### Protocol

**Figure 3:**
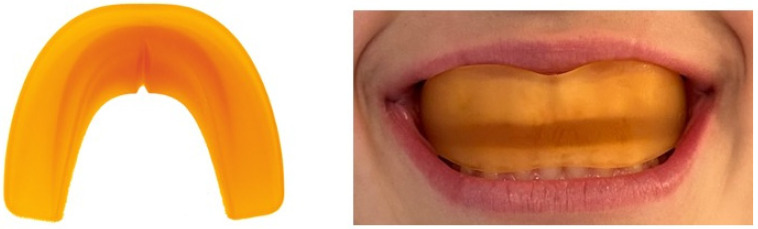
The Equilibrator Eptamed 00 orange and the Equilibrator 00 orange in the oral cavity of the patient. As shown in the photo, the balancer embraces both arches and the upper part with the curvature is positioned at the level of the upper labial frenulum.

Test group patients were treated with an orange Eptamed Equilibrator (00 series) as shown in Figure 3 of medium hardness and adapted to the patient according to the shape of the dental arches. This device has a shape similar to a mouth guard, and encompasses both arches, reaching to the patient’s most distal molars. Several sizes are available and are adaptable to the arches and measured according to the distance between the palatal cusps of the upper first premolars or the corresponding deciduous molars. The device is activated by the bite, depending on soft elastic forces generated by muscular contraction. It is a removable functional appliance that, through the tooth repositioning guides and thanks to its elasticity, is able to rebalance the altered functions of the mouth, transmitting to the teeth the reset input necessary to arrange themselves in a balanced manner along the arches. The idea of using this technique stems from the fact that there are no longer any annoying and unsightly iron rods, but rather a flexible device that can be removed at any time, mainly used at night. Thanks to its shape and consistency, it can rebalance the entire temporomandibular joint with enormous aesthetic and postural benefits.

The equilibrator is worn overnight and it exploits the power of lingual resting at the spot in the expansion of the palate. The balancer is an orthodontic device that encourages growth and, through muscle movement input, stimulates tissue development toward proper chewing function. Biting this elastomeric device equilibrates strain at the level of the sphenobasilar synchondrosis, based on osteopathic practice and philosophy. The mechanism of operation of elastodontic devices is such that, through the more or less elasticity of the material, a force is generated that can intervene three-dimensionally within a reality that is also three-dimensional such as the oral cavity. Moreover, thanks to an upper and lower canal they can accommodate the upper and lower teeth by guiding their correct position in the arch. In addition, there is a lingual ramp or internal slide that stimulates the positioning of the tongue on the palate, which in turn induces an increase in the transverse diameters of the upper arch, encourages nasal breathing, relaxes the orofacial musculature, relaxes the fascial musculature and harmonises phonation. 

**Figure 4:**
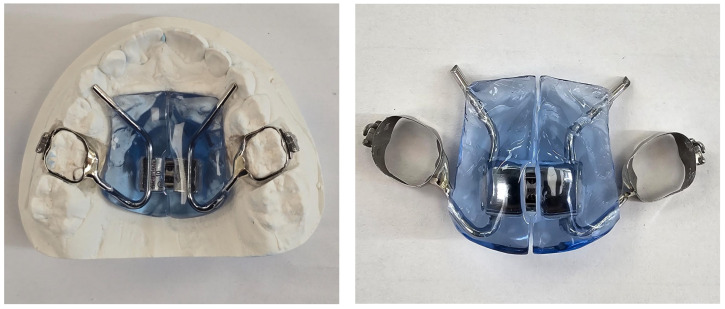
The Haas expander. As shown in the figure, the bands are placed on deciduous teeth.

Subjects in the test group were instructed to wear the device overnight and to present for monthly follow-up visits. Control group patients, on the other hand, were treated with a Haas expander with bands placed on their first upper permanent or deciduous molars, as shown in Figure 4. The use of the Haas expander is recommended at this age in cases of mono- or bilateral cross-bite, and band placement on deciduous teeth is often preferred. This allows a reduction in periodontal damage, attachment loss, reduced enamel demineralization, and external root resorption of permanent molars [14]. Haas devices were cemented to patients in the control group and were chair-activated with two quarter-turns (0.40 mm) by the orthodontist (Lamparski protocol 2003). Parents were instructed on home activation of the two-quarter-turn screw every day (0.40 mm) for 15 days and were then rechecked by the orthodontist every two weeks. Once the desired expansion was reached, the Haas device was blocked and left in situ for 3 months. All measurements of the two groups were recorded at T0 (before the start of therapy) and at T1 (12 months later). All patients were collaborative. No enrolled patients dropped out of therapy.

## 3. Results

For statistical analysis, the *t*-test for continuous variables and chi-square test for categorical variables (sex) were used for the two groups. Statistical significance was set at *p* < 0.05. As shown in Table 1, at baseline, the demographic and clinical characteristics of the two groups were considered. In particular, there is no statistical difference related to the sex or age of subjects in the two groups at all stages. In fact, all the values are not statistically significant for *p* < 0.05, the value related to sex is 1, and the value related to age is 0.143. Also, the mean and SD related to IMWand ICW at T0 and T1 are not statistically significant. 

**Figure 5:**
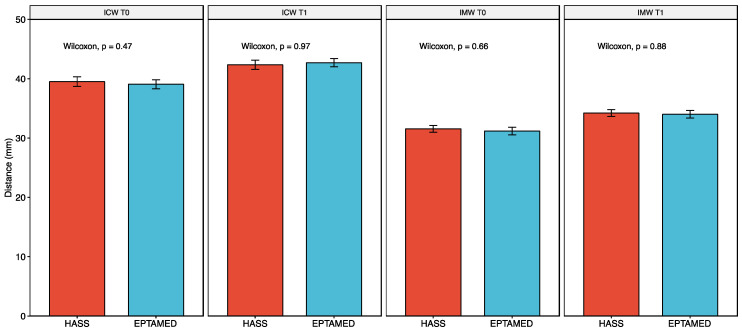
Results of Wilcoxon test analysis. In the figure, there is a Barplot of the different values stratified by timing according to group “Eptamed” vs. group “HAAS”. The two groups exhibited similar results that are not statistically significant.

To ascertain whether any pre-treatment and post-treatment differences in the two widths between the two groups were related to gender and physiological growth, the differences between IMW and ICW values were evaluated with the nonparametric Wilcoxon sum-rank test for conservativeness. Therefore, the results depended only on the type of device used. The two devices were found to be equally effective. No statistically significant differences were found between the expansions obtained with the Haas and Eptamed devices at T0 and T1. The two devices have similar effectiveness, as shown in Figure 5; the bar chart depicted therein can highlight how the two devices (Hass in red, Eptamed device in blue) are similar in all results.

**Figure 6:**
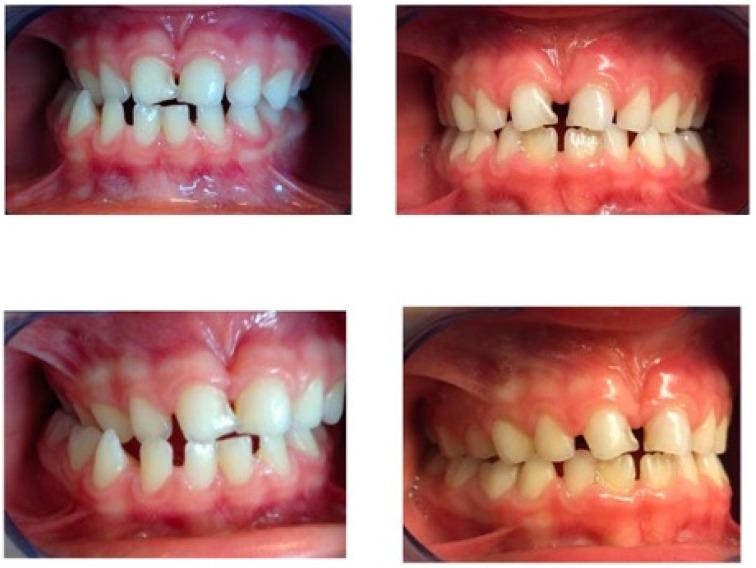
Intra-oral photos of a patient treated with the Eptamed device.

Photos of patients treated with the Eptamed device are reported in Figure 6.

## 4. Discussion

The results of the present study showed the following:

− intercanine distance increases less than intermolar distance in both groups
− the two devices would appear to work equally in resolving transverse discrepancies

**Figure 7:**
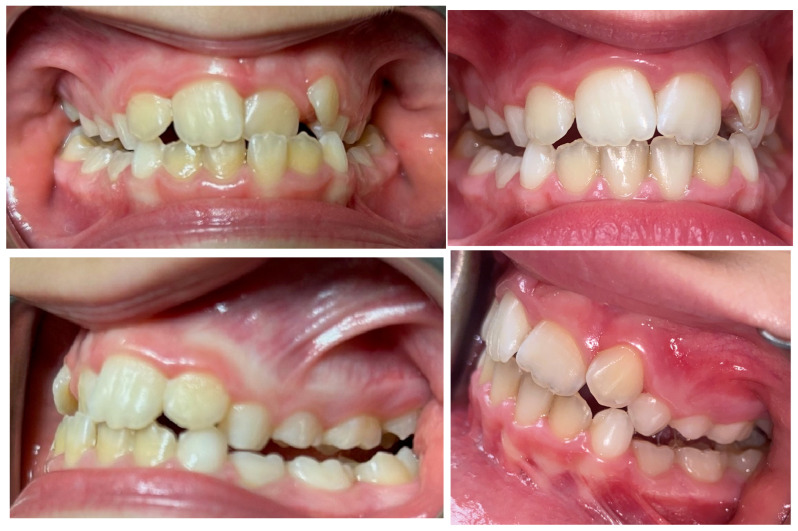
Intra-oral photos of a patient treated with the Haas Expander.

This study showed that the two devices were similarly effective for treatment of upper discrepancies, regardless of sex and age (Figure 6 and Figure 7). Several previous studies have compared the effectiveness of different fixed or removible devices in transverse palatal expansion and their results are similar to these [15]. It wascompared the treatment of posterior cross bite with removable and quad-helix transverse expansion plates. Although the plates required longer treatment than the quad-helix devices, the results were similar, with an improvement in transverse discrepancy in both groups. In another similar study, it was seen that the most common complications that can occur during palatal expansion, such as appliance breakage, were less frequent during treatment with removable plaque than during treatment with quad-helix. However, treatment duration and costs were higher in the expansion plate group than in the quad-helix group. The reason why, in our study, the intermolar distance would seem to increase more than the intercanine distance would seem to be related to the fact that the bands anchored on the posterior teeth would promote enlargement more [16,17]. Boysen et al. and Petren et al., in their interesting studies, evaluated the slow expansion performed with the quad-helix vs. that performed with transverse plates, and in both papers it was found that the intermolar distance increased more than the intercanine distance [18,19]. The opposite results emerged in the study by Bjerklin et al., in which the intercanine distance increased more than the intermolar distance with the same devices (quad-helix and palatal plates) [20]. 

Lippold et al. compared rapid expansion by bonded Hyrax appliance with a group of untreated patients, and in their analysis it was found that intercanine and intermolar distance values increased more in the test group than in the untreated group [19]. The same results were obtained by Lo Giudice et al., who studied the use of elastodontic devices in transverse discrepancies compared with untreated subjects. Intermolar width (IMW) and intercanine width (ICW) increased significantly in patients with this device in the mouth [11]. On the other hand, studies by Idris et al. found discordant results with those of our study. In fact, modified activators were found to be more effective than myofunctional devices (T4k, Trainer for Kids) in resolving class II division I malocclusions, which was also correlated with less pressure, tooth sensitivity, and pain [21].

However, there are no articles in the literature comparing the results obtained with elastodontic appliances and traditional orthopaedic appliances. Nowadays, new imaging techniques enable clinicians to assess three-dimensional anatomic changes very precisely and accurately. La Blonde et al. retrospectively compared changes in height and thickness of the maxillary bone attained by using two different activation protocols (0.5 mm per day test group and 0.8 mm per day control group) for rapid palatal expansion with a Hyrax-type expander, and investigated whether faster expansion could cause more adverse effects (e.g., alveolar and dental tipping, fenestration, and dehiscence). Palate width, palatal and buccal cortical bone thickness, alveolar bone height, angulation, and root length were measured using 3D imaging software (Dolphin Imaging Software 11.7 Premium). Palate width and buccal-lingual tooth angulation increased in both groups, but to a greater degree in the 0.8 mm per day activation protocol; in addition, buccal alveolar height and width decreased significantly in both groups, but more so with 0.8 mm per day activation. Increased transverse dimensions of the arches were observed in both groups, and were greater in the group treated with device activation of 0.8 mm per day, but were also associated with increased tooth tipping and greater reduction in vestibular bone cortical thickness [22]. Furthermore, in another study comparing arch dimensions following the use of RME and an MME, these devices achieved very similar results, as in our study. According to that study, RME and MME can be considered two effective treatment options for improving transverse arch dimensions and gaining space in the dental arches [23]. The above results can be compared with those of our study. To our knowledge, routinely-used palatal expanders have not been compared to novel devices such as the Equilibrator. Our study indicated that palatal expansions attained by the two devices were similar.

Elastodontic devices, however, act at the craniosacral level by facilitating and accompanying the development of the arches according to the correct growth vectors. Here, we tried to appreciate how elastodontic devices may correct palatal discrepancies, and thereby improve general health. In a study conducted by Ortu et al., electromyographic analysis of patients wearing elastodontic devices demonstrated a reduction in muscle tension in those with mandibular retrusion. These devices are simple to use and comfortable, can be worn only at night and for a few hours during the day, they don’t have metal clasps or wires, but are simple silicone products, all of which ensure greater adherence to therapy with lower costs for both the operator and the patient. They remove spoiled habits of the stomatognathic system and are able to act within the oral cavity on the teeth but also at the mucosal level, improving breathing, swallowing, and postural abnormalities.

These devices have a morphology that allows them to aid the natural expansion of the arches due to the natural re-education of the perioral and masticatory musculature, avoiding the need for orthopedic treatment (maxillary skeletal expansion) in those individuals with mild maxillary arch constriction. Moreover, due to an accentuated lingual ramp, they encourage the tongue to reach the palatine spot. Tongue contact at the palatine spot enables proper palate development and functional swallowing in children. Actually, various other studies have found that elastodontic devices are able to act even in several cases of malocclusion, in cases of anterior dental crowding, overjet, and overbite [24]. Moreover, elastodontic devices turn out to provide greater muscle relaxation, even and especially in patients with temporomandibular disorders and in need of orthodontic treatment, thus preventing symptoms from worsening [25]. Due to the elastic properties of the material from which these devices are made, trauma to the gums and oral mucosa is hardly reported, and at the same time the balancers are strong enough to withstand chewing loads [26]. We hypothesize that the risk of recurrence may be much higher in Haas-treated than elastodontic-treated patients because Haas does not directly impact the functioning of the stomatognathic system (breathing, tongue position, centripetal activity of the perioral muscles). Moreover, as Haas acts through rapid palatal expansion, while reducing treatment time, there are several disadvantages: pain and discomfort from the excessive forces required to open the palatine suture, parental involvement in activating the appliance, and the likelihood of appliance breakage. Rapid palatal expansion with such devices was considered to be the most painful orthodontic procedure of all for 98% of patients undergoing orthodontic treatment in childhood [26]. On the other hand, the dento-alveolar expansion exerted by elastodontic devices, despite needing longer treatment times, reduces the aforementioned negative effects of rapid expansion. Finally, compared with Haas, these removable appliances allow the patient to maintain proper oral hygiene, reducing gingival inflammation and the growth of plaque and tartar that occurs with Haas devices mainly due to the presence of orthodontic bands [13]. 

However, the results obtained from this study need to be analyzed very carefully. Our study, in fact, is a pilot study and as such has several limitations. The sample size is indeed small; we analyzed only 64 patients. It would be advisable to increase the sample size so as to further strengthen the results obtained. Moreover, the time period in which these patients were seen is limited, re-evaluations were carried out only after 1 year from the start of therapy. Again, it would be appropriate to increase the follow-up period. It is deemed necessary in the future to repeat this study with a larger and more homogeneous cohort and for a longer period of time.

## 5. Conclusions

The aim of this article was to verify the clinical changes in the upper arch during the expansion with two different devices. However, elastodontic devices are more comfortable during the resolution of palatal discrepancies. The enhancement of dynamic function addresses the cause of the malocclusion, minimizes the risk of relapse, and thus maintains stable occlusion over time with fewer concerns for the orthodontist. In the end, these removable appliances, worn during the night and functioning without activations such as repositioning, do not cause pain. Dental movements can be evaluated and recorded by using novel digital dentistry devices: using a simple intraoral scan, the orthodontist can plan individualized therapy, amend tooth movements, document progress, and assess the true area of transverse expansion of the palate.

## Figures and Tables

**Table 1:** Results (mean and SD) of the *t*-test for continous variables and chi-square test for categorical variables (sex) for the two groups. The IMW and ICW values at T0 and T1 are expressed in mm. There is no statistically significant difference in the values of the groups related to sex or age at all stages.

		EPTAMED	HAAS	*p* Value
*n*		32	32	
Sex (%)	F	16 (50.0)	16 (50.0)	1
	M	16 (50.0)	16 (50.0)	
Age (mean (SD))		7.08 (0.44)	7.32 (0.50)	0.143
IMW_T0 (mean (SD))		31.19 (2.61)	31.56 (2.31)	0.670
IMW_T1 (mean (SD))		34.00 (2.56)	34.22 (2.29)	0.800
ICW_T0 (mean (SD))		39.06 (3.00)	39.50 (3.20)	0.693
ICW_T1 (mean (SD))		42.69 (2.77)	42.34 (3.09)	0.743

## Data Availability

The data presented in this study are available on request from the corresponding author. The data are not publicly available due to privacy.
